# Intracranial lipoma with extra cranial subcutaneous component with a midline bony defect and persistent falcine sinus

**DOI:** 10.1259/bjrcr.20220011

**Published:** 2023-07-22

**Authors:** Santh Kumar Bellamkonda, Balamuralikrishna Vadana, Chandrasekhara rao Kondragunta, Swetha Balije

**Affiliations:** 1 Department of Radiodiagnosis, Dr Pinnamaneni Siddhartha Institute of Medical Sciences & Research Foundation, Chinnavutupalle, India

## Abstract

Intracranial lipomas are only 0.06–0.46% of intracranial lesions, forming a rare type of congenital malformation. Interhemispheric lipoma associated with subcutaneous component is extremely rare. They are usually asymptomatic, but may also present with seizures, raised intracranial pressure, dementia, hemiparesis, persistent headaches, psychomotor retardation and cranial nerve defects. These are associated with vascular, bone, tentorial and other abnormalities. MR examination must be considered to evaluate for a possibility of intracranial component and to rule out other anomalies. Here, we present features of a rare presentation of intracranial lipoma.

## Clinical presentation

**Figure 1.**
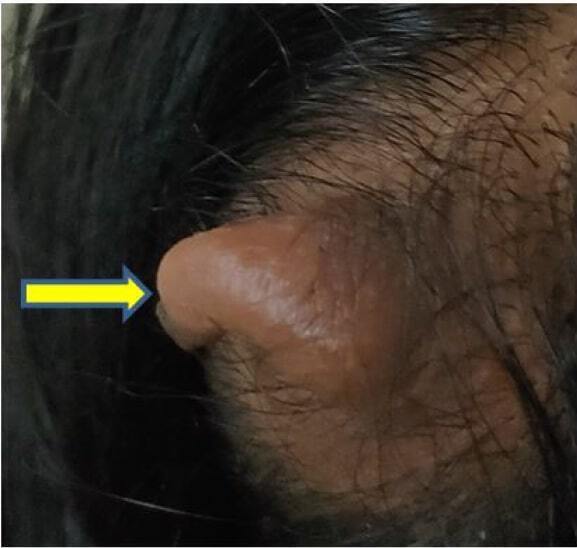


A 25-year-old female presented with a midline soft scalp mass which is noticed at birth was referred to our hospital. On physical examination,^1–3^ a soft exophytic mass measuring 4 × 3 cm is present in the midline at parietal region of scalp (Figure 1). Nervous system examination and other system examination are normal. Other routine investigations were normal.

**Figure 2.**
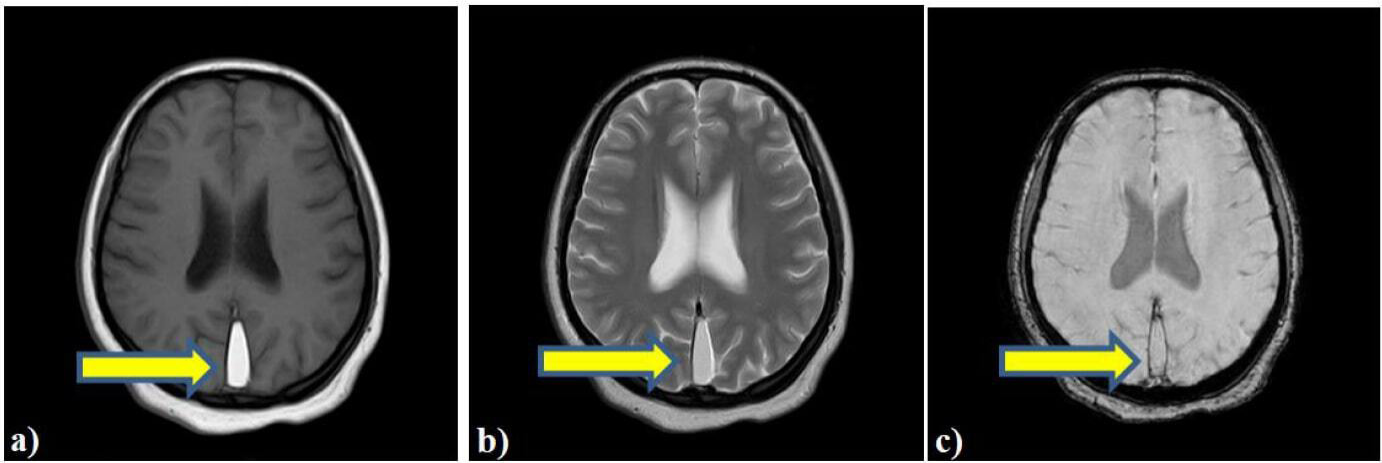
**a**) axial T1W image; **b**) axial T2W image showing midline interhemispheric hyperintense mass at parietal region; **c**) axial SWI showing mild peripheral succeptibility blooming surrounding the lipoma.

**Figure 3.**
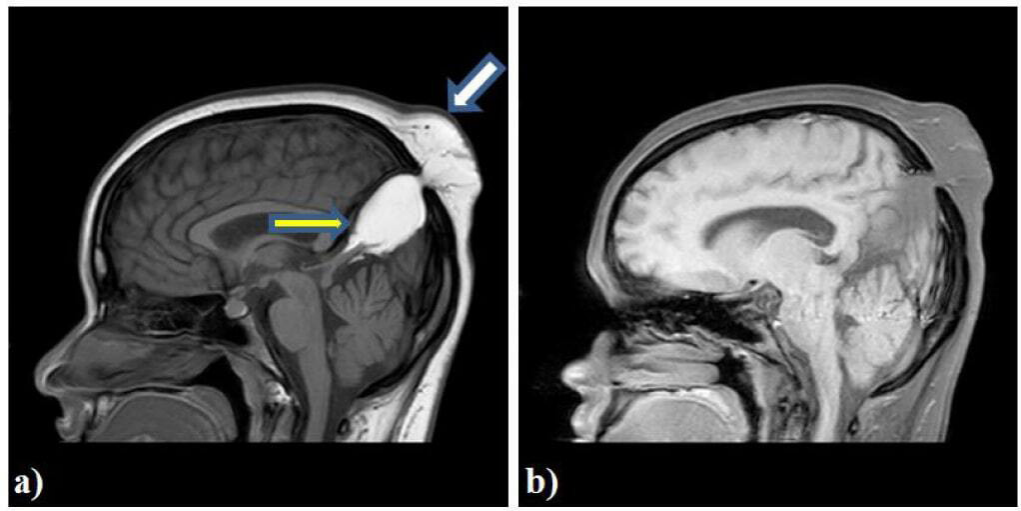
**a**) sagittal T1W image showing a interhemispheric hyperintense mass (yellow arrow) ith a extracranial extension (white arrow) into the scalp subcutaneous tissue through a midline bony defect at parietal bone. Acute tectal beaking noted into the mass; **b**) sagittal fat-saturaed T1W image showing hypointense mass at interhemisphereic region confirming fat component.

On MR imaging, a posterior interhemispheric mass lesion measuring 5.0 × 2.5 × 1.2 cm is seen posteroinferior to the superior sagittal sinus. It is hyperintense on *T*_1_-weighted image and hypointense on fat-suppressed *T*_1_-weighted image which is consistent with lipoma (Figure 2). Parasagittal portion of the tentorium is extending superiorly upto falcine sinus and acute tectal beaking into this lipoma noted (Figure 3).

**Figure 4.**
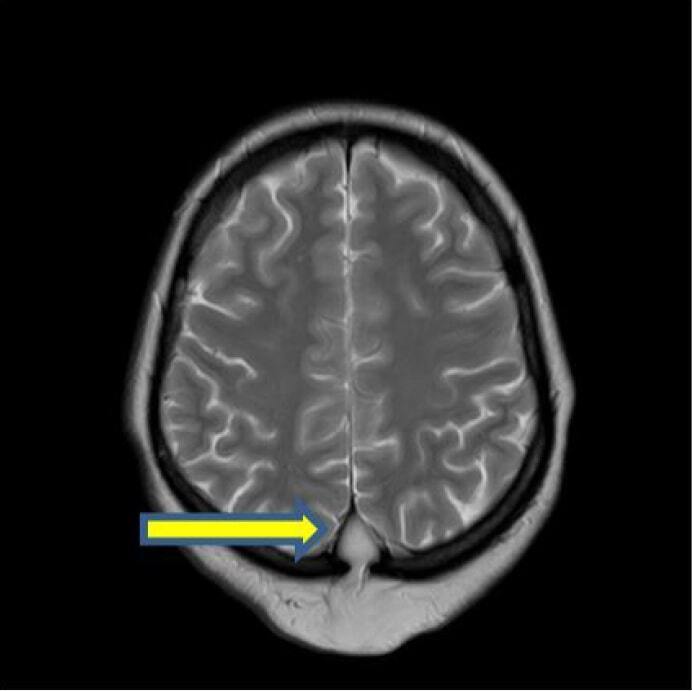
Axial T2W image showing hyperintense mass at interhemisphereic region showing a extracranial extension through a defect in the parietal bone and a connecting stalk (yellow arrow).

The lesion is showing extracranial extension into subcutaneous plane of scalp through a small midline bony defect measuring 5 × 5 mm in sagittal plane at parietal bone level (Figure 4). This extracranial component is showing increased vascularity.

**Figure 5.**
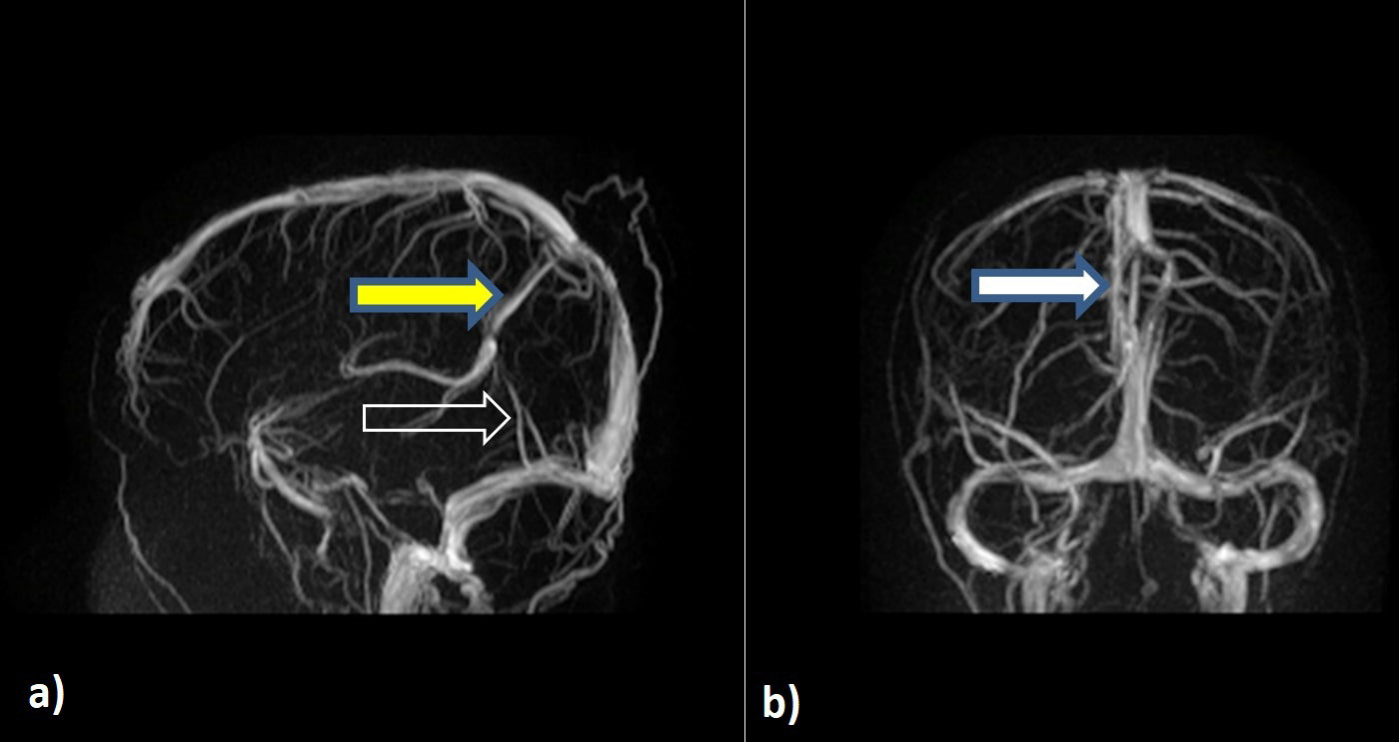
**a**) Sagittal MRV showing persistent falcine sinus (yellow arrow) draining into superior sagittal sinus. Absent straight sinus (open white arrow); **b**) coronal MRV showing superior sagittal sinus fenestration (white arrow) at the level of lipoma.

On MR venography, the absence of straight sinus is seen with normal torcular position. Fenestration in the sagittal sinus is seen beneath the subcutaneous lipoma which corresponds with the defect in the parietal bones. Anterior to this fenestration persistent falcine sinus is seen in the midline draining into the superior sagittal sinus (Figure 5).

## Discussion

Intracranial lipomas are 0.06–0.46% of intracranial lesions, forming a rare type of congenital malformation.^1^

Interhemispheric lipomas are the most common type about 45% and the others are quadrigeminal or superior cerebellar (25%), suprasellar or interpeduncular (14%), cerebellopontine angle (9%) and sylvian (5%).^2^

Several pathological hypotheses for development of intracranial lipomas are: (a) at the time of the neural tube formation, the faulty disjunction of cutaneous ectoderm from overlying mesoderm; (b) preexisting meningeal fatty tissue hypertrophy; (c) meningeal connective tissue transformation or metaplasia; (d) maldifferentiation of the abnormally persistent meninx primitiva into lipomatous elements.^3^

It is extremely rare to have a interhemispheric lipoma with a extracranial subcutaneous component.^1^ The connection between the two components varies from a tiny track or stalk to one bulky, continuous lesion.^4^

In case of midline facial dysraphism, there is a small finger like evagination of meninx primitive through the future anterior cranium secondary dehiscence, through which the intracranial component will be contiguous with the subcutaneous component of the lipoma. It could be isolated if the vault defect closes, excluding the two embryologically related lipoma segments.^1^ In our case, the defect is not closed and the intracranial component is connected by a stalk to the extracranial component through the defect in the cranial vault.

Most of the patients are asymptomatic, but may also present with persistent headaches, hemiparesis, seizures, raised intracranial pressure, cranial nerve defects, dementia and psychomotor retardation.^2,3^

Association with anomalies like lipomas at other sites, corpus callosum dysgenesis, absent septum pellucidum, hypoplastic fornix, vermis agenesis, myelomeningocele, encephaloceles, spina bifida, bone defects, heterotopic gray matter, and cleft lip are seen. Anomalies are severe if the insult occurs at early stage of organogenesis.^1^

A variety of vascular abnormalities like arterial and venous narrowing, kinking and distension, engulfment of the cerebral arteries, arteriovenous malformation and cerebral arteries aneurysms are seen.^1^ But our case had the absence of straight sinus and vertically oriented persistent falcine sinus within the lipoma running through the sagittal sinus fenestration which is not reported ever.

Approximately on the 35th embryonal day, transverse and superior sagittal sinuses appear as the primitive marginal sinus. Approximately on 50th day of embryogenesis, right and left primitive marginal sinuses join together in the midline. This is followed by superior sagittal sinus formation in midline at approximately 10 weeks of gestational age.^5^ In our case, the interhemispheric lipoma which is extending to subcutaneous plane of scalp in the midline is developed mostly before the fusion of primitive marginal sinuses forming a fenestration in the superior sagittal sinus by the stalk which is connecting both the components.

The falcine sinus is a embryonic vascular structure which is also called as a precursor of straight sinus. It develops from the mesenchyme at the level of the mesencephalic flexure. It usually closes after birth but can be found later in life in conditions like straight sinus underdevelopment and dysplastic tentorium cerebri.^6^ In our case, the absence of straight sinus is mostly due to interference in its development by interhemispheric lipoma or due to the insult that lead to the formation of lipoma. This lead to persistence of the falcine sinus as a alternative venous drainage.

The primary cause for abnormalities in vascular, tentorial and bone is due to mesenchymal disturbance. During the eighth and ninth weeks of gestational age, two centers of ossification for each parietal bone appear and later ossify in a membranous pathway. At 3 months of intrauterine life, the tentorium is formed by the coalescence of right and left tentorial membranes that fuse in the midline.^6^ In our case, there is a defect in the parietal bone and tentorial beaking is also seen at the level of lipoma. Along with the falcine sinus persistence, the absence of the straight sinus and fenestration in the superior sagittal sinus along with bone defect at the same level is mostly due to mesenchymal disturbance leading to all these defects.

Treatment is mostly by conservative management. Surgical resection can be attempted. Complications include engulfment of vital structures like cranial nerves and blood vessels due to its adhesive property.^4^

## Conclusion

In conclusion, intracranial lipoma with subcutaneous component is a very rare pathology. Infants who have subcutaneous scalp lipoma, MR examination must be considered to evaluate for a possibility of intracranial component and to rule out other anomalies and for assessment of vascularity which is important when a surgical procedure is contemplated.

## Learning points

1. Intracranial lipomas appear hyperintense on *T*_1_-weighted MR imaging and less hyperintense on *T*_2_-weighted imaging. Chemical shift artefact can be observed with the larger lesions, particularly on *T*_2_-weighted sequences. Suppression of signal within the lipoma on STIR (short tau inversion recovery) or fat-saturation sequences confirms the presence of fat within the lesions. No enhancement on contrast study.
2. Usually associated with other anomalies and these anomalies are severe if the insult occurs at early stage of organogenesis.
3. The primary cause for abnormalities in vascular, tentorial and bone is due to mesenchymal disturbance.
